# Palatal Rugae Patterns in Edentulous Cases, Are They A Reliable Forensic Marker?

**Published:** 2015-09

**Authors:** R. Poojya, C. S. Shruthi, Vaishali Mysore Rajashekar, Aswathy Kaimal

**Affiliations:** Department of prosthodontics, M. R. Ambedkar dental college and hospital, 1/36 cline road, cooke town, Bangalore, India

**Keywords:** Edentulous cases, forensic marker, forensic odontology, palatoscopy, palatal rugae patterns

## Abstract

One of the main objectives of the forensic sciences is establishing a person’s identity which can be a very complex process. The analysis of the teeth, fingerprints and DNA evaluation are probably the most used techniques allowing fast and secure identification processes. Palatal rugae or transverse palatine folds are asymmetrical and irregular elevations of the mucosa located in the anterior third of the palate and are permanent, prominent and unique for individuals and thus can be used as identification for forensic purposes widely in edentulous patients wherein no teeth are present in the oral cavity. In forensic odontology dentists play a prime role in supporting legal and criminal issues. Palatoscopy or palatal rugoscopy is the name given to the study of palatal rugae in order to ascertain a person’s identity. Studies have demonstrated that no two individual rugae patterns are alike in their configuration and the characteristic rugae pattern of the palate does not change as a result of growth. Hence this article reviews the significance of palatal rugae patterns in edentulous cases as a reliable forensic marker.

## INTRODUCTION

Transverse palatine folds or palatal rugae, are asymmetrical and irregular elevations of the mucosa situated in the anterior third of the palate, made from lateral membrane of the incisive papilla, arranged in transverse direction from palatine raphe located in the midsagittal plane. These formations have been used in medico legal identification processes as their individual morphological uniqueness are stable over time (1).

When a victim has no teeth at all in the oral cavity, it is termed as completely edentulous situation. In such cases, information for use in personal identification based on methods available in forensic odontology is much more limited than in cases of dentate victims where in teeth are present. For edentulous victims, some recognition methods are available, such as comparing the anatomy of the paranasal sinuses (2) and comparing the bony patterns seen on radiographs (3). As the victims denture themselves can provide us with more personal information with regard to denture materials, their unique shapes, for use as ante mortem data or postmortem evidence is considered.

Among the evidence taken from an edentulous victim a palatal rugae pattern is one of the distinctive and relatively obtainable morphological features and the patterns can be taken not only directly from the hard palate but also from the mucosal surface of the dentures. Application of palatal rugae patterns to personal identification was first suggested by Allen in 1889 (4).

Physiologically the palatal rugae are involved in the oral swallowing and help to progress the relationship between food and the taste receptors in the dorsal surface of the tongue and also participate in speech and in suction in children (5).

Palatal rugae form elevations more or less prominent and take various configurations. Its design and structure are unaffected and are not altered by chemicals, heat, disease or trauma (6). Form, layout and characteristics are not affected by eruption of the teeth or their loss, but sometimes palatal rugae adjacent to the alveolar arch slightly change their position after tooth extraction (7). However some events may contribute to changes in the pattern of palatal rugae such as finger sucking in childhood and persistent pressure due to orthodontic treatment. Furthermore it has been reported that extraction can make a local effect on the direction of the palatal rugae (8).

**Figure 1:**
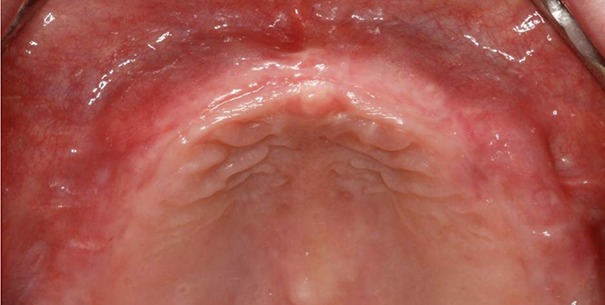
Palatal rugae patterns in edentulous cases.

Palatal rugae have been considered relevant for human identification due to its stablility (9) being equivalent to the finger print which is unique for each person. The anatomical position of the rugae inside the mouth surrounded by cheeks, lips, tongue, and buccal pad of fat, teeth and bone keeps them well protected from trauma and high temperatures. Thus, they can be used consistently as a reference landmark during identification (10). It can be of particular interest in edentulous cases and also in certain conditions where finger prints cannot be taken, such as burnt bodies or where bodies have undergone decomposition. By identification of the rugae pattern a Prosthodontist may identify the possessor of upper denture (11) (Figure 1).

In forensic odontology dentists play a major role in sustaining legal and criminal issues (12). Palatoscopy or palatal rugoscopy, is the name given to the study of palatal rugae in order to establish a person’s individuality (13). Studies have demonstrated that no two individual’s rugae patterns are alike in their arrangement and the characteristic rugae pattern of the palate does not change as a result of growth (14).

Kuppler, in 1897 was the first person to study palatal anatomy to identify racial anatomic features. In 1937, Carrea developed a detailed study and established a way to classify palatal rugae. In 1946 Martins dos Santos presented a practical classification based on rugae location. In 1983, Brinon following the studies of Carrea, divided palatal rugae into two groups (fundamental and specific) in a similar way to that done with finger prints.

## ANATOMICAL ASPECTS

**Figure 2:**
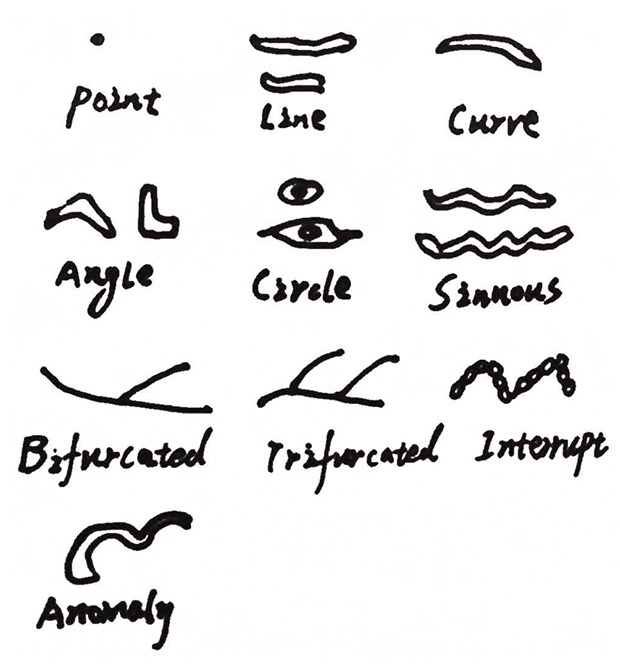
Martin dos Santos palatal rugae classification.

Palatal rugae are formed in the 3^rd^ month in utero from the hard connective tissue covering the bone. Once formed they do not experience any changes except in length, due to normal growth. Investigations have been done on the thermal effect and the decomposition changes on the palatal rugae of burn victims and stated that most victims did not sustain any palatal rugae pattern changes. Furthermore, the capability of palatal rugae to resist decomposition changes for up to seven days after death was noted (Figure 2).

## ANALYZING AND RECORDING PALATAL RUGAE

Palatal rugae once formed, is unique for every individual. There are several ways to analyse the palatal rugae. Intraoral inspection is probably the most used method as it is easy and economical. A more detailed and accurate method is by use of photographs and by making impressions.

Oral photography or oral impressions can be used to analyze palatal rugae. Stereoscopy, can obtain a three dimensional image of palatal rugae anatomy. It is based on examination of two pictures taken with the same camera, from two different points using special equipments. Superimposition of various digital photographs for comparing rugae patterns can be performed using various computer software. E.g, RUGFP-ID, palatal rugae comparison software (PRCS version 2.0) Calcorrugoscopy or overlay print can be used to perform comparative analysis. Sterophotogrammetry which by using special device called traster marker allows for correct determination of length and position of every single rugae.

Gitto et al. described a method where palatal rugae were added to a complete denture in order to get better speech patterns in some patients. This process can lead to false identity exclusion due to misleading ante mortem data (15).

Denture marking or labeling is not a new concept in either prosthetic or forensic dentistry, and its routine practice has been urged by forensic dentists internationally for many years. The supervising authority on the health sector should make denture marking compulsory and as Prosthodontists, we request our colleagues, other dental specialists, and general dental practitioners that it is the professional and ethical duty of ours to do so.

One of the methods of denture labeling is engraving. This system involves marking the models during manufacture so that denture carries the marked information. A label marker is used to prepare an embossed plastic pattern, which in turn is used to insert a marked metal plate in to denture framework. This technique is simple and facilitates amalgamation of a stable and fire proof label in the denture base material (16).

## DISCUSSION

Palate is fornix shaped and trapped by dental arch. There is a thin, longitudinal mucosal protrusion in the median line of hard palate, it is actually three-seven soft tissue ridges radially evoked from incisive papillae and median palatal suture, and its shape is irregular and asymmetric (17).

Palatal rugae protrusions are mainly dense connective tissue, and palatal rugae developed can be triggered by local epithelial cell proliferation. Palatal rugae pattern is determined by continuous fibers concentrically around the palate. The fibroblasts and collagen fibers accumulate in the thickening connective tissue below epithelial cells, presenting different directions. The core fibers in the human palatal rugae contain many factors to maintain the palatal shape. The main component of palatal rugae is hydrophilic glycosaminoglycan which enhances tissue swelling and helps maintain palatal pattern (18).

Identification of rugae can be mislead by poorly demarcated eminences of the rugae, in edentulous cases were the most common cause of difficulty in identification in forensic studies. In cases with severely low eminences ,the shape or form of alveolar arches are not very clear and it may be difficult to compare in such situations .Secondly change of palatal height mainly caused by atrophy of the alveolar bone after losing the teeth is an added cause of low rate of accuracy, when tracings of the rugae are used to compare patterns in edentulous cases. Thirdly non complex patterns such as straight rugae, with well demarcated eminences sometimes led to trouble in establishing identification.

Camargo et al. have referred that in gingival graft surgery, the selection of the palatal donor site should avoid the rugae areas because they may continue in the grafted tissues (19).

Palatal rugae stability is considered an important factor when teeth are extracted as has been demonstrated in several studies, which points out the stability of the rugae medial points over the lateral points. In addition to this, it has been said that more posterior rugae are less vulnerable to changes with tooth movement. The third palatal rugae pair in particular the most stable reference (20).

Limson and Julian have developed a computer software program which makes use of the principle frequently employed in finger print analysis. The method used digitalized images of the palate on which characteristic points were plotted on the medial and lateral extremities of all rugae. The plotted points were assessed by the software programme and the information stored sequentially corresponding to pixel position. These researchers obtained up to 97% accuracy in recognizing individuals in simulated post and ante mortem comparison of the palatal rugae.

A recent study by obtam and coworkers states that high precision rates in postmortem identification from palatal rugae can be obtained by straight forward visual comparison of post and ante mortem rugae patterns obtained from dentures.

The curve and sinous types are dominantly observed in men or women (men: curve 21.43%, sinuous 24.13%; women: curve 18.28%.sinous 22.7%) it is also noted that men presented with more rugae than women. Furthermore there were more palatal rugae at the right side compared with left side.

In a study done by Saraf, rugae pattern can help to discriminate between Indian male and female. This study did not show any major difference in length of rugae, whereas rugae shape had implication on sex differentiation (21).

Palatal rugae patterns can differentiate the features among populations, because palatal rugae pattern and distribution are unique in each person .On the other hand large scale relative study concerning different races, family members and monozygotic twins is urgently needed in the future. In addition, we should propose uniform standards and procedures for palatal rugae pattern collection, recording and computer analysis, which is advantageous to the establishment of palatal rugae systems in forensic identifications.

## CONCLUSION

Palatal rugae is of prime importance, and can be used as a reliable guide in forensic identification. Among the proof taken from an edentulous victims, palatal rugae pattern is one of the unique and relatively available morphological features and the patterns can be taken not only directly from the hard palate but also from the mucosal surface of the dentures. A forensic odontologist must have broad background knowledge of general dentistry encompassing all dental specialties. This branch needs auxiliary research and recognition in India.
